# Diffusion weighted magnetic resonance imaging demonstrates tumor response following palliative embolization of a recurrent shoulder plasmacytoma

**DOI:** 10.1186/1477-7819-12-271

**Published:** 2014-08-22

**Authors:** Viktor Bérczi, Gábor Rudas, Lajos Rudolf Kozák, Tamás Györke, Gábor Mikala, Tamás Masszi, Ildikó Kalina, Pál Novák Kaposi

**Affiliations:** Department of Radiology and Oncotherapy, Semmelweis University, Üllői út 78/a, H-1082 Budapest, Hungary; MRI Research Center, Szentágothai J Knowledge Center, Budapest, Hungary; Department of Nuclear Medicine, Semmelweis University, Budapest, Hungary; Department of Haematology and Stem Cell Transplantation, St Istvan and St Laszlo Hospital, Budapest, Hungary; Scanomed Ltd, Budapest, Hungary; Third Department of Internal Medicine, Semmelweis University, Budapest, Hungary

**Keywords:** plasmacytoma, transcatheter arterial embolization, DWIBS, MRI, PET-CT, tumor response, bortezomib, palliative treatment

## Abstract

We report the palliative embolization and functional imaging follow-up of a recurrent shoulder plasmacytoma. The multiple myeloma patient complained of severe pain and discomfort, while he could not tolerate further chemotherapy. The left shoulder lesion had earlier received a high dose of irradiation. Thus, the well-vascularized lesion was embolized via feeding arteries branching off from the left subclavian artery in two sessions. The patient’s symptoms rapidly improved post-embolization and the serum free light chain ratio stabilized at a lower level. The follow-up magnetic resonance image showed increased diffusivity in previously restricted tumor foci. This has negatively correlated with the decreased fludeoxyglucose uptake on PET, suggesting post-embolization necrosis.

**Electronic supplementary material:**

The online version of this article (doi:10.1186/1477-7819-12-271) contains supplementary material, which is available to authorized users.

## Background

The first results for the selective transcatheter arterial embolization (TAE) of bone tumors were reported in 1975 [1]. Since then, TAE has been successfully used to control symptoms in patients with either primary or metastatic disease. Nevertheless, TAE does not have a role in the standard treatment of plasma cell lesions. In the few reports available, TAE has been performed in selected cases of solitary bone plasmacytomas to reduce the risk of bleeding during surgical resection [2, 3]. Here we report that TAE promptly reduced pain and discomfort in a patient with a recurrent plasma cell tumor in the left shoulder. We chose TAE because the patient had already received the maximum dose of local irradiation and could not tolerate further chemotherapy. Functional studies, including diffusion weighted magnetic resonance imaging (MRI) with background subtraction (DWIBS) as well as fludeoxyglucose positron emission tomography (^18^FDG-PET), are recent additions to the imaging of multiple myeloma (MM) [4, 5]. We utilized both techniques during the follow-up, and detected excellent conspicuity of the post-embolization response.

## Case presentation

**Figure 1:**
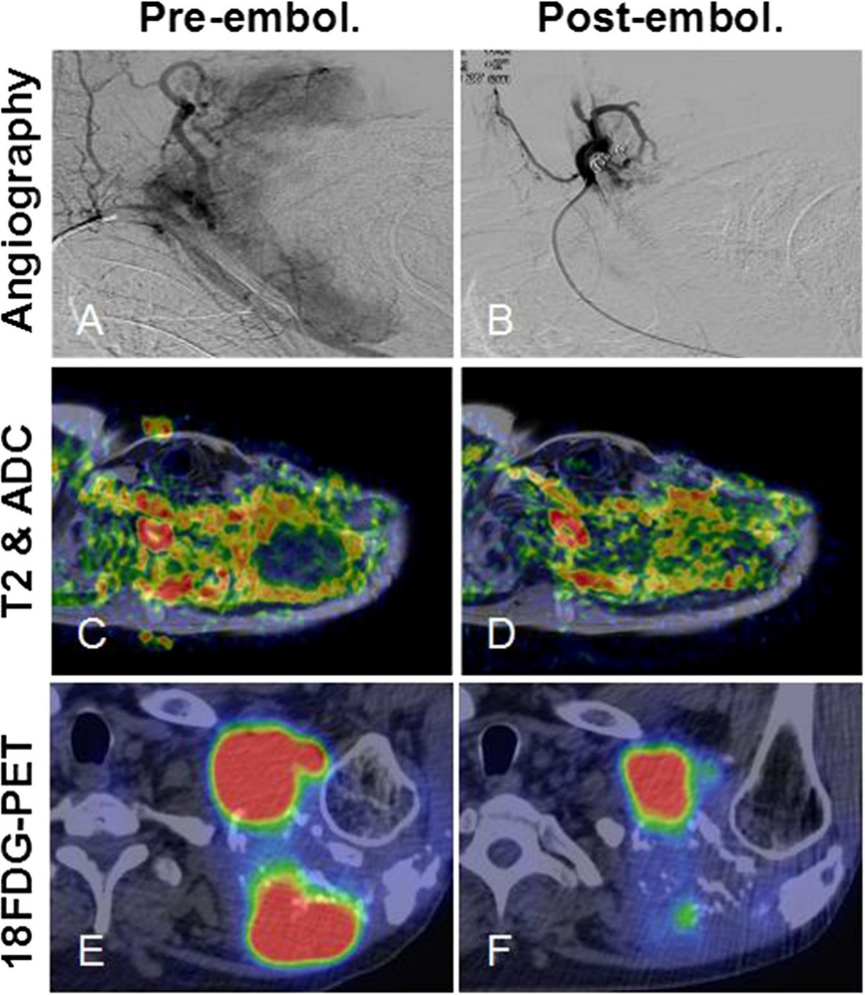
**Post-embolization tumor response in the shoulder plasmacytoma could be best demonstrated with functional imaging studies. (A)** Angiogram of the left shoulder revealed a well-vascularized lesion, which was supplied by branches from the left subclavian artery. **(B)** Selective catheterization of the feeding arteries and embolization of the tumor vasculature with 350 to 500 μm PVA particles were performed in two sessions from a right femoral puncture and a left brachial puncture, respectively. Some arterial branches were also closed off with coils. DWIBS MRI proved to be a sensitive modality for locating densely packed foci of tumor cells inside a heterogeneous lesion. **(C)** We co-registered ADC maps with T2 weighted images that allowed more precise comparison between baseline and follow-up scans. **(D)** The increase of ADC values by 4 weeks post-embolization was clearly visible on the fusion map. **(E)** Some DWIBS restricted foci also showed high ^18^FDG uptake on PET-CT. **(F)** Following embolization, decreased metabolic activity was detected, which was inversely correlated with increased diffusivity, a sign of tumor necrosis.

A 58-year-old man was first diagnosed with solitary plasmacytoma of the left scapula 19 years ago. At the time he was treated with radiation therapy. The tumor had recurred multiple times, for which a total dose of 38 Gy of local irradiation was administered. A second lesion occurred in the right acetabulum. Afterwards, the patient was treated with multiple cycles of combination chemotherapy, and twice with autologous stem cell transplantation (Additional file 1: Table S1). In spite of all therapeutic efforts, the disease did not go into full remission. During the last episode of recurrence, after two cycles of effective salvage with a VDT-PACE combination (bortezomib-dexamethason-thalidomide-cisplatine-doxorubicine-cyclophosphamide-etopozide), the regimen had to be stopped due to the sustained thrombocytopenia.The patient continued to complain of significant pain, and restricted movements of the upper limb. Computed tomography (CT) and MRI scans showed a multifocal tumor in the left scapula, which extended into the axilla thus, surgical resection was impossible. At this point, we decided to use TAE to achieve fast control of the patient’s symptoms. Angiography showed the lesion was well vascularized (Figure 1A). Embolization of the lesion was performed in two sessions; feeding arteries branching off from the left subclavian artery were selectively catheterized from a right femoral puncture and a left brachial puncture, and the tumor vessels were embolized to stasis (Figure 1B) with 350 to 550 μm Contour® PVA particles (Boston Scientific, Natick, MA, USA). Intravenous pethidine was administered for analgesia. The platelet count was 161 K at the time of the procedure. We neither observed bleeding nor any neurological deficit in association with the embolization. The post-procedural period was uneventful; and the patient was released to home on the next day.

The baseline and the consecutive MRI examinations were performed on a 3 T Philips Achieva scanner (Philips Healthcare, Eindhoven, the Netherlands) using DWIBS (*b* = 0, 800, 1300) and contrast enhanced sequences. The lesion’s size remained stable after the embolization. Meanwhile, DWIBS showed increasing diffusivity in some previously diffusion restricted tumor foci (Figure 1C,D). We co-registered ADC maps with T2 weighted images, and identical regions of interest (ROI) were drawn around representative tumor areas for quantitative analysis (Additional file 2: Figure S1). PET-CT showed low ADC areas had overlapped with metabolic hot spots and some ^18^FDG avid foci lost tracer uptake post-embolization. Interestingly, there was a negative correlation between decreasing metabolic activity and increasing diffusivity in these areas (Figure 1E,F), indicating cell loss. Serum electrophoresis showed monoclonal free light-chain κ (FLCκ) levels had also regressed (Table 1).

**Table 1:** **Results of serum electrophoresis**

Clinical status	Follow-up time (weeks)	FLCκ (mg/l)^b^	***k***/λ ratio^b^
Baseline	0	4570	4009
Chemotherapy^a^	11	145	NA
Pre-embolization	28.5	913	78.7
Post-embolization	31	349	8.18
Control	35	350	36.57

The characteristic free kappa light-chain (FLCκ) level returned close to the normal range after embolization.

^a^The test was taken following two cycles of VDT-PACE chemotherapy.

^b^The normal range is 3.3 to 19.4 mg/l for FLCκ and 0.26 to 1.95 for the *k*/λ ratio.

At follow-ups, the patient reported only moderate discomfort and occasional use of pain medication, and remained relatively symptom free for 6 months. Unfortunately, after temporary improvement, the plasmacytoma became again symptomatic and required additional chemotherapy by 14 months.

## Conclusions

Plasma cell tumors are highly radiation sensitive; thus, radiotherapy is the primary choice of treatment [6]. Occasionally, surgery is combined with radiotherapy to prevent pathological fractures, or to remove a lesion completely. Distant bone lesions and bone marrow involvement may indicate progression to MM. These cases require systemic chemotherapy or stem cell transplantation. Although palliative TAE can be performed for both primary and metastatic bone tumors, this technique is rarely used to treat myeloma lesions. In a couple of cases, solitary bone plasmacytomas have been embolized pre-operatively to reduce bleeding [2, 3].

In our patient, the left shoulder region had already received a high dose of irradiation. Combination salvage chemotherapy had to be stopped after the second cycle as the patient developed severe thrombocytopenia and polyneuropathy. The tumor diffusely infiltrated the scapula as well as the axilla; therefore, a surgical consultation deemed the lesion non-resectable.

We contemplated that TAE could provide quick local control of the patient’s symptoms. Embolization with PVA microspheres is a well-established palliative technique. The rationale of selective TAE is to inject small thrombogenic particles into tumor arterioles causing ischemic necrosis and reducing micro-bleeding, thus reducing symptoms [7]. An angiogram showed a well-vascularized tumor suitable for embolization. TAE was also readily available in our institution, the risk of complications was minimal and embolization did not preclude other therapeutic efforts.

The results of the embolization were apparent on radiologic follow-ups. The lesion’s size remained stable. Meanwhile, a partial response was detected with functional studies. MRI is a frequently used morphologic imaging modality for MM specifically, to detect bone-marrow involvement. DWIBS is a sensitive tool, which can identify highly cellular myeloma lesions, as they characteristically show restricted diffusion [4]. According to a recent study, ADC values in skull tumors inversely correlate with cellularity and could be used to differentiate between malignant and benign bone lesions [8]. We found multiple diffusion restricted foci inside the shoulder lesion and hypothesized that these corresponded to nests of viable plasma cells inside a heterogeneous tumor, although histological samples were not available for confirmation. Average pre-treatment ADC values in these foci ranged between 0.7 and 1.0 × 10^−3^ s/mm^2^ at 3 T, which is similar to malignant lesions of the skull, and to metastatic lesions in MM [4, 5, 8]. Horger *et al*. described that DWIBS is not only able to locate intramedullary foci in MM, but can also detect a short-term response following chemotherapy [4]. Messiou *et al*. reported that ADC values showed a significant increase for MM bone lesions 4 to 6 weeks after systemic treatment [5]. We confirm that DWIBS can be used to monitor the post-embolization response in plasma cell lesions, as we observed significantly increased ADC values in multiple foci. Embolization-induced cell death and cellular dehydration are the most likely explanation for the observed diffusivity changes.

^18^FDG PET is considered the most reliable method for detecting early treatment response in MM [9]. Interestingly, we found a negative correlation between decreased FDG uptake and increased diffusivity in some tumor areas on the follow-up scans. We speculate that the ischemia resulted in cell loss, cellular dehydration and low glucose uptake following embolization. Similar results were reported in a preliminary study by Byun *et al*., who compared ^18^FDG-PET with diffusion-weighted MRI following neoadjuvant therapy for osteosarcoma [10]. They found that SUV and ADC were negatively correlated, while PET and diffusion-weighted MRI predicted the histological response with similar accuracy.

Clinically, the patient’s symptoms significantly improved following TAE. The decreased serum immunoglobulin ratio indicated a reduction in the tumor mass. This was in part the results of the embolization; however, previous chemotherapy could have also contributed to the systemic effect. Importantly, the TAE achieved temporary control of symptoms for almost 6 months. During this period, the bone marrow reserve could recover, neurotoxicity diminished and administration of salvage chemotherapy become possible for inducing remission again. In summary, TAE can relieve the acute pain and discomfort of plasmacytomas. The sensitivity of DWIBS is comparable to ^18^FDG-PET for detecting an early tumor response post-embolization.

## Consent

This case report has been approved by the Institutional Review Board of the Department of Radiology and Oncotherapy, Semmelweis University. Written informed consent was obtained from the patient.

## Electronic supplementary material

Additional file 1: Table S1: Summary of the anti-myeloma treatment administered prior and after the palliative embolization. (DOC 35 KB)

Additional file 2: Figure S1: **(A)** Changes of diffusivity could be compared in identical ROIs on the pre- and post-embolization MRI scans after ADC maps were co-registered with T2 weighted images using anatomic landmarks. **(B)** Quantitative analysis of the diffusivity maps showed that the ADC values followed a normal distribution, and were significantly elevated post-embolization in the shoulder area. **(C)** No significant change in diffusivity could be demonstrated in another focus in the axilla. (JPEG 91 KB)

## Abbreviations

^18^FDG: fludeoxyglucose
CT: computed tomography
DWIBS: diffusion weighted magnetic resonance imaging with background subtraction
FLCκ: free light-chain κ
MRI: magnetic resonance imaging
PET: positron emission tomography
TAE: transcatheter arterial embolization.
