# Supplementary material for: Diffusion weighted magnetic resonance imaging demonstrates tumor response following palliative embolization of a recurrent shoulder plasmacytoma
Source: World J Surg Oncol. 2014 Aug 22;12:271. doi: 10.1186/1477-7819-12-271 (PMC4150974; doi:10.1186/1477-7819-12-271)
Supplement: Supplementary file 1 — Additional file 1: Table S1: Summary of the anti-myeloma treatment administered prior and after the palliative embolization. (DOC 35 KB) [file 12957_2014_1745_MOESM1_ESM.doc]

**Additional file 1: Table S1** Summary of the anti-myeloma treatment administered prior and after the palliative embolization.

| **Date** | **Important plasmacytoma/myeloma related medical event** |
| --- | --- |
| MAY/1994 | Diagnosis of plasmacytoma is the scapula  30 Gy local irradiation |
| 1996 | Additional 3 Gy of palliative irradiation to residual shoulder plasmacytoma |
| 1997 | Pelvic plasmacytoma and multiple myeloma diagnosed  24 Gy irradiation to pelvic plasmacytoma  5 Gy irradiation to residual shoulder plasmacytoma  6 cycles of VAD (vincristine, doxorubicine, dexamethason) chemotherapy |
| APR/1998 | Cyclophosphamide for stem cell mobilization  High-dose melphalan for conditioning + autologous stem cell transplantation |
| 2006 | Relapse of myeloma  3 cycles of PID (bortezomib, idarubicine, dexamethason) chemotherapy |
| 2008 | Relapse  4 cycles of bortezomib-dexamethason therapy |
| 2009 | Relapse  4 cycles of AD (doxorubicine, dexamethason) chemotherapy  V-EDAP (bortezomib, etopozide, dexamethason, cisplatine, cytosine arabinozide) chemotherapy for stem cell mobilization  High-dose melphalan for conditioning + autologous stem cell transplantation |
| NOV/2010 | Relapse  2 cycles PADT (bortezomib,doxorubicinem dexamethason, thalidomide) chemotherapy |
| MAY/2011 | Relapse  2 months cyclophosphamide-dexamethason palliation |
| JUL/2011 | Relapse  3 cycles VDT-PACE (bortezomib-dexamethason-thalidomide-cisplatine-doxorubicine-cyclophosphamide-etopozide) chemotherapy |
| JAN/2012 | Relapse  **Transarterial embolization** |
| MAR/2013  JUL/2013 | Relapse  3 cycles VD-Benda (bortezomib-dexamethason-bendamustine) chemotherapy  Patient is again in remission |
