# Supplementary figures and images for: Diffusion weighted magnetic resonance imaging demonstrates tumor response following palliative embolization of a recurrent shoulder plasmacytoma
Source: World J Surg Oncol. 2014 Aug 22;12:271. doi: 10.1186/1477-7819-12-271 (PMC4150974; doi:10.1186/1477-7819-12-271)

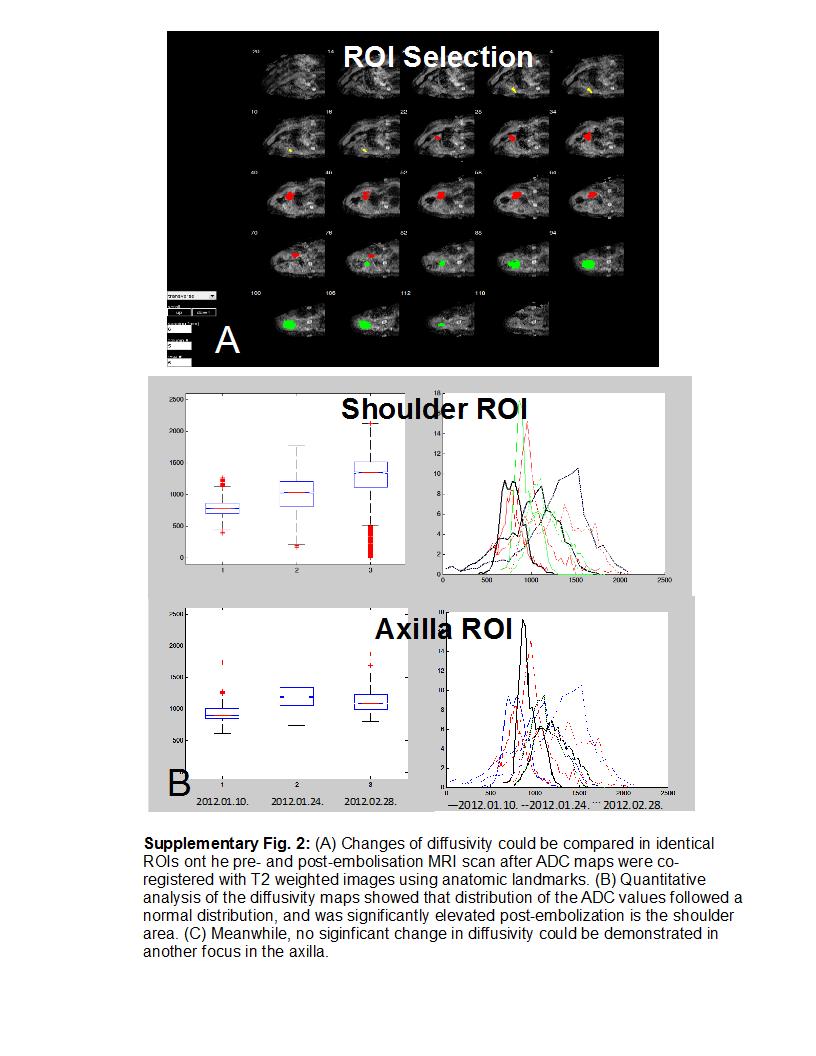

Supplement: Supplementary file 2 — Additional file 2: Figure S1: (A) Changes of diffusivity could be compared in identical ROIs on the pre- and post-embolization MRI scans after ADC maps were co-registered with T2 weighted images using anatomic landmarks. (B) Quantitative analysis of the diffusivity maps showed that the ADC values followed a normal distribution, and were significantly elevated post-embolization in the shoulder area. (C) No significant change in diffusivity could be demonstrated in another focus in the axilla. (JPEG 91 KB) [file 12957_2014_1745_MOESM2_ESM.jpeg]
